# Eosinophilic gastritis and gluten-sensitive enteropathy manifested as hypoproteinemia and treated with omalizumab: a case report

**DOI:** 10.1186/s13223-024-00878-8

**Published:** 2024-03-05

**Authors:** Zhirong Du, Zixi Wang, Weixun Zhou, Jia Yin, Yuxiang Zhi

**Affiliations:** 1 Department of Allergy, Chinese Academy of Medical Sciences and Peking Union Medical College, Beijing Key Laboratory of Precision Medicine for Diagnosis and Treatment of Allergic Disease, National Clinical Research Center for Dermatologic and Immunologic Diseases, Peking Union Medical College Hospital, No. 1 Shuaifuyuan Street, Wangfujing, Beijing, 100730 China; 2 Department of Pathology, Peking Union Medical College Hospital, Chinese Academy of Medical Sciences, Beijing, China

**Keywords:** Eosinophilic gastritis, Gluten-sensitive enteropathy, Hypoproteinemia, Omalizumab

## Abstract

**Background:**

Eosinophilic gastritis (EoG) has rarely been reported in conjunction with gluten-sensitive enteropathy (GSE). When this does occur, patients typically present with gastrointestinal symptoms. To our knowledge, hypoproteinemia has not been reported as the primary manifestation. Anti-IgE therapy, such as omalizumab, lowers eosinophil counts in the blood, lungs, and gut. Its efficiency in treating active EoG remain unknown.

**Case presentation:**

We report a 33-month-old boy with a history of food allergy and atopic dermatitis who developed recurrent edema, hypoproteinemia, and eosinophilia at the age of 14 months. The diagnoses of EoG and GSE were confirmed based on the clinical presentation and results of gastrointestinal biopsies and serological testing. Although prednisone and dietary intervention were initially effective, the boy developed prednisone-related facial swelling. After stopping prednisone, his symptoms relapsed. Subsequent treatment with omalizumab, combined with dietary intervention, showed good efficacy and safety.

**Conclusions:**

To our knowledge, this is the first case of concurrent EoG and GSE that presented primarily with hypoproteinemia. We highlight the rare manifestations of these two diseases to raise clinical suspicion and prevent missed and delayed diagnoses. The pathogenesis of EoG is heterogeneous and complex. Omalizumab showed good efficacy, indicating that IgE-mediated processes may be involved in the pathogenesis of this patient’s diseases.

## Background

Eosinophilic gastrointestinal diseases (EGIDs) are inflammatory disorder characterized by eosinophilic infiltration of the gastrointestinal tract [1]. Eosinophilic esophagitis (EoE) is the most common EGIDs. Non-EoE EGIDs include eosinophilic gastritis (EoG), eosinophilic enteritis, and eosinophilic colitis (EoC). The constellation used to be termed eosinophilic gastroenteritis (EoGE) and is rare, with a prevalence of 5.1/100,000 [2]. Gluten-sensitive enteropathy (GSE) is an immune-mediated disorder caused by the consumption of gluten, and 2.19% of adolescents and young adults in China are positive for serum markers of GSE [3]. GSE and EoG rarely occur together. Corticosteroids are the mainstay of treatment for EoG [4]. However, considering the adverse effects of long-term corticosteroid use and refractory status, other therapies, including omalizumab, have been explored [4]. Currently, a strict gluten-free diet is the only available treatment option for GSE [5]. Here, we report the first case of EoG and GSE with hypoproteinemia as the main clinical manifestation that was successfully treated with omalizumab combined with dietary intervention.

## Case presentation

A 33-month-old boy with a history of atopic dermatitis (AD) presented to our clinic complaining of recurrent edema, hypoproteinemia, and eosinophilia since the age of 14 months. He experienced a local skin rash immediately after facial exposure to cow’s milk at 12 months of age. He had been exclusively breastfed and had never ingested cow’s milk. His father had a history of allergic rhinitis, and his mother had a history of acute urticaria.

At 15 months of age, laboratory examination showed elevated peripheral blood eosinophils (1.21 × 10^9^/L), decreased albumin levels (17.3 g/L; normal range 35–52 g/L), and decreased globulin levels (12.4 g/L; normal range 20–40 g/L). Food allergen-specific immunoglobulin (Ig) E testing (ImmunoCAP system, Phadia AB, Uppsala, Sweden) indicated multiple food allergies including egg white (80.1 kU_A_/L), cow’s milk (16.7 kU_A_/L), soybean (15.0 kU_A_/L), peanut (14.9 kU_A_/L), wheat (50.6 kU_A_/L), shrimp (0.47 kU_A_/L), and fx1 nut mix (peanut, hazelnut, Brazil nut, almond, and coconut) (14.6 kU_A_/L). We performed a skin prink test (SPT) for food allergens (using a prick-to-prick test). The SPT results for cow’s milk, egg, wheat, and gliadin were positive (Table 1). Initial gastroscopy revealed congested and edematous mucosa of the gastric antrum and slightly rough mucosa of the duodenal bulb and descending portion. A biopsy specimen obtained from the esophagus was normal. Biopsy specimens obtained from the antrum revealed mild chronic gastritis with a slight increase in the number of eosinophils scattered throughout the lamina propria (7–28 / high power field (HPF)), while biopsy specimens obtained from the descending part of the duodenum revealed mild chronic active enteritis. *Helicobacter pylori* test results were negative, as was the 99mTc-HSA scan result. The patient underwent further extensive evaluation, including stool tests for parasites, parasite-specific antibodies, antinuclear antibodies, antineutrophil cytoplasmic antibodies, chest CT, bone marrow aspiration, and biopsy to rule out parasitic, autoimmune, and hematological diseases.

**Table 1:** Skin prick test results

Test agents	Wheal, mm × mm	Erythema, mm × mm
Histamine	6 × 3	10 × 10
Saline	0 × 0	0 × 0
Cow’s milk	3 × 4	20 × 15
Egg white	10 × 15	20 × 15
Egg yolk	6 × 4	15 × 7
Peanut	0 × 0	0 × 0
Soybean	2 × 2	10 × 10
Wheat	7 × 4	10 × 10
Gliadin	4 × 7	20 × 10
Sea shrimp	0 × 0	0 × 0
Sea crab	0 × 0	0 × 0

The patient was diagnosed with a food allergy and EoG. Potential food allergens (egg, milk, soybean, peanut, wheat, shrimp, and nuts) were temporarily avoided, even though he did not experience any obvious immediate allergic symptoms such as rash, cough, abdominal pain, and diarrhea upon ingestion of foods other than milk. Dietary interventions showed no benefits. The patient’s symptoms improved after supplementation with albumin (6 g per day for 10 days) and gamma globulin (2.5 g per day for 5 days), but relapsed quickly after completing the treatment. The patient was then treated with prednisone, cetirizine, and montelukast along with the same dietary intervention, and his symptoms improved.

However, at 19-months of age, 1 month after the discontinuation of prednisone, the peripheral blood eosinophils increased to 2.97 × 10^9^/L, and the albumin levels decreased to 32.4 g/L. A repeat gastroscopy showed that the esophageal mucosa was congested and rough, with furrow-like changes, and the dentate line was not clear. The mucosa of the gastric fundus, body, horn, and antrum was hyperemic with rough and blotchy changes. In addition, scattered ulcers and erythema were detected in the gastric body and antrum, respectively. Esophageal biopsy revealed an increased number of eosinophils in the epithelium and lamina propria (17–40 / HPF). Biopsies of the gastric body and antrum revealed eosinophilic gastritis with active lesions and increased eosinophilic infiltration of the epithelium (3–50 / HPF, 6–50 / HPF). A duodenal biopsy showed mild active inflammation of the small intestinal mucosa. Prednisone (5 mg twice daily) was reinstituted, and the peripheral blood eosinophil and albumin levels returned to normal. Prednisone was gradually tapered and stopped after six months due to the patient developing facial edema.

**Fig. 1:**
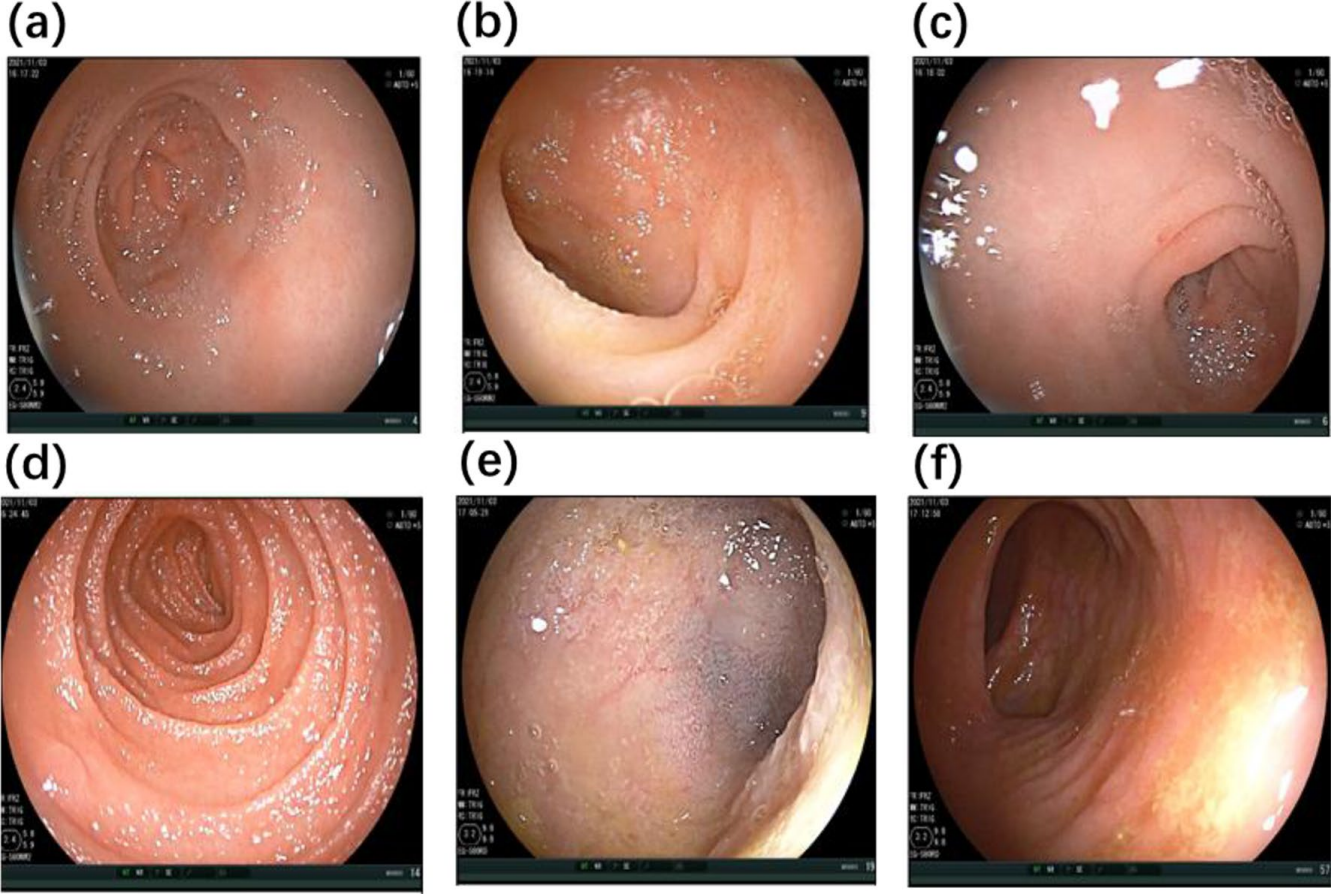
Gastroenteroscopy findings in this patient. (**a**) Striped and flaky red mucosa in the gastric antrum. (**b**) Scattered and flaky red mucosa in the duodenal bulb. (**c**) Scattered and flaky red mucosa in the junction of the descending duodenum. (**d**) White and raised mucosa (4 × 4 mm) in the horizontal part of duodenum. (**e**) Unevenly distributed villi at the end of the ileum. (**f**) Mucosal edema in descending colon

**Fig. 2:**
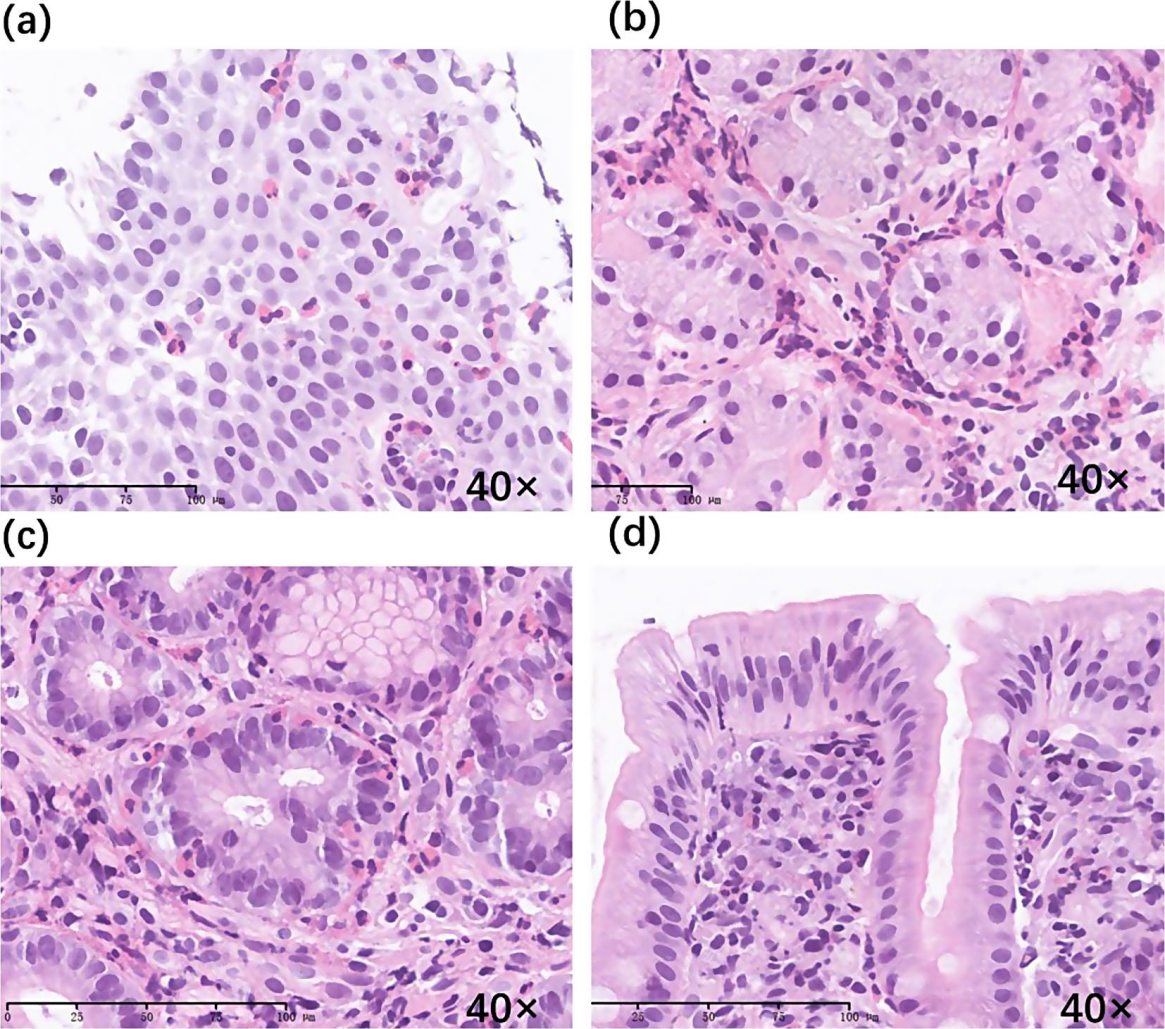
Pathological findings in this patient. (**a**) Esophageal pathology showing chronic inflammation of the squamous epithelial mucosa and eosinophil infiltration in the epithelium. (**b**) Gastric body pathology showing mild chronic inflammation of the gastric mucosa and increased infiltration of eosinophils, especially in the deep layer of the mucosa. (**c**) Gastric antrum pathology showing mild chronic inflammation of the gastric mucosa, increased infiltration of eosinophils, and partial infiltration of the epithelium. (**d**) Duodenal bulb pathology showing blunt contraction, flattening of the small intestinal villi, and lymphocyte infiltration in the superficial epithelium. Hematoxylin and eosin (H&E) staining, magnification × 40

Unfortunately, at 24 months of age, eyelid edema recurred 9 days after prednisone discontinuation. The peripheral blood eosinophil count increased to 3.38 × 10^9^/L, and albumin levels decreased to 28.2 g/L. Subsequent evaluation revealed positive results for anti-tissue transglutaminase (tTG) antibodies (IgA 87.69 U/ml, IgG 37.87 U/ml; normal range 0–20 U/ml), anti-endomysial antibodies (EMA) (IgA 123.42 U/ml; normal range 0–20 U/ml), and deamidated gliadin peptide (DGP) (IgA 50.03 U/ml, IgG 18.18 U/ml; normal range<10 U/ml). Gastroenteroscopy showed a 3 × 3 mm white and slightly raised mucosa in the greater curvature of the gastric body; scattered and flaky red mucosa in the gastric antrum, duodenal bulb, and junction of the descending duodenum; 4 × 4 mm white and raised mucosa in the horizontal part of the duodenum; unevenly distributed villi at the end of the ileum; and mucosal edema in the descending colon (Fig. 1). Biopsy specimens from esophagus revealed increased eosinophilic infiltration of the epithelium. Biopsy specimens from the antrum, showed eosinophil increased and infiltrated partly into the epithelium. Biopsy specimens from the gastric body, showed increased eosinophilic infiltration, especially in the deep layer of the mucosa. Duodenal biopsies from the horizontal part showed few small intestinal villi. Biopsies from the duodenal bulb showed chronic and acute inflammation, eosinophil infiltration in the lamina propria (40 / HPF), obvious atrophy of the villi, and lymphocyte infiltration in the superficial epithelium (Fig. 2). The patient was diagnosed with EoG and GSE.

A strict gluten-free diet was initiated, and potentially food allergens (egg, milk, soybean, peanut, shrimp, and nuts) were completely avoided. Montelukast and cetirizine were then administered. Simultaneously, budesonide atomization was administered for two weeks to treat an upper respiratory tract infection. His edema was completely ameliorated, the albumin levels increased to normal, and the peripheral blood eosinophil count decreased to 1.15 × 10^9^/L. However, one month later, the symptoms recurred. Except for budesonide atomization, the same interventions were used, but showed no effect. Infliximab was administered three times (100 mg/dose) but was ineffective in resolving the patient’s edema. Laboratory tests showed elevated peripheral blood eosinophils (2.7 × 10^9^/L), decreased albumin levels (19 g/L), and decreased globulin levels (11.6 g/L). IgG levels (2.73 g/L; normal range 3.5–5.5 g/L) were decreased, while IgA and IgM levels were normal. Anti-tTG IgA, anti-tTG IgG, anti-DGP IgA, and anti-DGP IgG tests were negative after the five-month gluten-free diet. The total serum IgE level was elevated (735 kU/L). Omalizumab was administered at 150 mg every two weeks for 16 weeks, and then at 150 mg every four weeks for 16 weeks. 21 weeks after the start of omalizumab therapy, peanuts, eggs, shrimp, soybeans, and nuts were gradually added into the diet and the patient could tolerate these foods other than eggs, which causes perioral rash and vomit. Cow`s milk caused a perioral rash and was still being avoided. During this period, the patient did not exhibit any edema. The albumin level remained normal, and the peripheral blood eosinophils count remained at 0.82–1.97 × 10^9^/L. Molecular diagnostics showed positive specific IgE to casein (Bos d 8) (4.83kU_A_/L), alpha-lactalbumin (Bos d 4) (8.54kU_A_/L), beta-lactoglobulin (Bos d 5) (23.5kU_A_/L), bovine serum albumin (BSA) (Bos d 6) (14.9kU_A_/L), ovomucoid (Gal d 1) (20.3kU_A_/L), ovalbumin (Gal d 2) (21.4kU_A_/L), and conalbumin (ovotransferrin) (Gal d 3) (7.99kU_A_/L). Specific IgE against cross-reactive carbohydrate determinants (CCD) was 1.15KU/L. Gastroscopic re-examination showed that the eosinophils in the gastrointestinal tissue decreased to normal levels, with isolated intraepithelial lymphocyte infiltrations, crypt hyperplasia, and villus atrophy.

## Discussion and conclusions

EoG is a rare disorder characterized by eosinophilic infiltration of the stomach, which falls under the umbrella term of non-EoE EGIDs [4]. The clinical presentation of non-EoE EGIDs depends on the site, extent, and layer depth of the affected tissue, according to which it is classified into mucosal, muscular, and subserosal variants [4, 6]. The most common symptoms of non-EoE EGIDs are abdominal pain, diarrhea, nausea, and vomiting [7]. Diffuse small bowel lesions may lead to protein-losing enteropathy, which is associated with severe morbidity [4]. Interestingly, the main clinical manifestation in the patient in this report was hypoproteinemia. Considering the possibility of false-negative 99mTc-HSA scans in children [8], protein-losing enteropathy was still highly suspected, despite the negative scan result in this case. Additionally, our patient had an elevated peripheral eosinophil count and co-existing allergic conditions, as well as biopsy evidence of eosinophilic infiltration of the esophagus, gastric antrum, and gastric body [7]. How to term EGID with esophageal and gastric involvement remains controversial and challenging [1]. EoG can be complicated with EoE, as well as result in esophagus involvement [9]. In this case, the esophageal lesions appeared after gastrointestinal lesions, and the patient had no “EoE-like” symptoms, so he was diagnosed with “EoG with esophageal involvement.”

Approximately 45–63% of patients with non-EoE EGIDs have a history of allergies, including food or drug allergies, rhinitis, asthma and eczema [10]. It was reported [11] that an amino acid–based elemental diet improves symptomatic, quality-of-life, histologic, endoscopic, and molecular parameters of EoG/EoGE; the study also found: disease recurrence with food trigger reintroduction support a dominant role for food allergens in disease pathogenesis. Given the heterogeneity of non-EoE EGIDs, food allergens may only play a dominant role in certain types of these diseases. Our patient had a definite milk and egg allergy and AD. Component tests showed that he could not tolerate heated milk or eggs either. In addition, the patient tested positive for specific IgE corresponding to multiple food allergens. Non-EoE EGIDs are suspected to result from food allergens that cross the intestinal mucosa and induce mast cell degranulation and eosinophil recruitment [10]. In this case, an elimination diet was administered, which is the initial therapy for non-EoE EGIDs; however, it showed no benefit. With the reintroduction of foods, such as shrimp, soybeans, and nuts, the patient’s condition remained stable. Given the presence of specific IgE against CCD, the patient did not need to avoid all positive food allergens. The underlying mechanisms of non-EoE EGIDs are still largely unclear, and the role of food allergy in non-EoE EGIDs is not as evident as in EoE, which needs to be further studied.

The patient in this case was also diagnosed with GSE with positive serological tests for anti-tTG, anti-EMA IgA, and anti-DGP antibodies. The anti-tTG and anti-DGP antibodies were negative after a 5-month gluten-free diet. Additionally, biopsy specimens revealed obvious atrophy of the villi and lymphocyte infiltration in the superficial epithelium of the duodenal bulb, confirming the diagnosis of GSE [12]. Clinical manifestations of GSE vary from malabsorption to asymptomatic individuals [12]. GSE can also cause protein-losing enteropathy [13]. Currently, a strict gluten-free diet is the only treatment option for GSE [5]. Although a gluten-free diet was initially effective when the patient was diagnosed with GSE, his symptoms later recurred despite adherence to a strict gluten-free diet.

Duodenal mucosal eosinophilia has been described in patients with GSE [14]. Both eosinophilic infiltration and extracellular eosinophilic major basic protein (MBP) deposition in small bowel biopsy specimens are significantly greater in patients with non-EoE EGIDs and GSE than in controls, suggesting that eosinophils play a role in these disorders through MBP [15]. Interleukin (IL)-3, IL-5, and granulocyte-macrophage colony stimulating factor, which are potent regulators of eosinophil recruitment, activation, and survival, have been detected in the jejunal mucosa of patients with GSE [16]. Serum IL-5 levels are significantly elevated in children with GSE compared to those in controls [17]. Therefore, the increased tissue eosinophil count observed in our patient may partly reflect the pathological changes secondary to GSE. However, no peripheral eosinophilia or increased eosinophil counts in parts of the intestinal mucosa other than in the duodenum have been observed in patients with GSE [14]. These findings suggest that the patient in our case may have had two separate diseases.

To the best of our knowledge, there are only two other reports of GSE and non-EoE EGIDs in the same patient [18, 19]. Interestingly, our patient presented with edema and hypoproteinemia rather than gastrointestinal symptoms. Similarly, a gluten-free diet combined with prednisone was effective for our patient. Increased levels of tumor necrosis factor (TNF)-α have been described in patients with non-EoE EGIDs, and TNF-α can lead to selective eosinophil recruitment by inducing cell adhesion molecules [4]. Infliximab, an anti-TNF-α monoclonal antibody, was proven to be effective for refractory EoC [4, 20]. For these reasons, our patient was treated with infliximab but did not show clinical remission. Abnormally increased eosinophil infiltration in the stomach and bowel is a key histopathological characteristic of non-EoE EGIDs [21]. Omalizumab, a humanized monoclonal antibody targeting the high-affinity IgE receptor, can reduce eosinophilia in the peripheral blood, bronchus, skin, and gut. A single-center study [22] demonstrated that omalizumab decreased peripheral eosinophilia and relieved symptoms in patients with EGID. However, the efficacy of omalizumab in the treatment of active EGID remains unclear. An allergic mechanism is postulated in non-EoE EGID. Half of patients with EoG tested positive for either food allergens or aeroallergens on SPT [23]. Omalizumab is effective in many allergic disorders, including asthma, allergic rhinitis, urticaria, and food allergy [24, 25]. Because this patient had high IgE levels and multiple food-specific IgE levels detected, we selected to use omalizumab for the treatment. Omalizumab therapy showed good efficacy, indicating that IgE-mediated processes may be involved in this patient’s disease pathogenesis. As the pathogenesis of non-EoE EGID is heterogeneous and complex, the effectiveness of omalizumab and who can benefit from it needs to be further studied.

Here, we report a rare case of simultaneously occurring EoG and GSE. EoG may be induced by food allergens [11], and GSE is induced by dietary wheat gliadin and related proteins [26]. The possibility of concurrent EoG and GSE should be considered in cases of unexplained hypoproteinemia, even in those without gastrointestinal symptoms. Each disease should be treated individually to achieve complete clinical remission. Omalizumab showed good efficacy in this case, indicating that IgE-mediated processes may be involved in this patient’s disease pathogenesis. As the pathogenesis of non-EoE EGID is complex, the efficiency of omalizumab and who can benefit from it needs to be further studied.

## Data Availability

The datasets used and/or analyzed during the current study are available from the corresponding author on reasonable request.

## Abbreviations

EGID: Eosinophilic gastrointestinal diseases
EoE: Eosinophilic esophagitis
EoG: Eosinophilic gastritis
EoC: Eosinophilic colitis
EoGE: Eosinophilic gastroenteritis
GSE: Gluten-sensitive enteropathy
AD: Atopic dermatitis
Ig: Immunoglobulin
SPT: Skin prink test
HPF: High power field
tTG: Tissue transglutaminase
EMA: Endomysial antibody
DGP: Deamidated gliadin peptide
CCD: Cross-reactive carbohydrate determinants
MBP: Major basic protein
IL-3: Interleukin-3
IL-5: Interleukin-5
